# Surface-Enhanced Raman Spectroscopy (SERS) Method for Rapid Detection of Neomycin and Chloramphenicol Residues in Chicken Meat

**DOI:** 10.3390/s25133920

**Published:** 2025-06-24

**Authors:** Yan Wu, Junshi Huang, Ni Tong, Qi Chen, Fang Peng, Muhua Liu, Jinhui Zhao, Shuanggen Huang

**Affiliations:** 1 Key Laboratory of Modern Agricultural Equipment in Jiangxi Province, Jiangxi Agricultural University, Nanchang 330045, China; wuyan1981@jxau.edu.cn (Y.W.); huangjunshi@jxau.edu.cn (J.H.); tongn@stu.jxau.edu.cn (N.T.); chenqi567726@jxau.edu.cn (Q.C.); peng5181@jxau.edu.cn (F.P.); lustertree@jxau.edu.cn (M.L.); zhaojh@jxau.edu.cn (J.Z.); 2 School of Computer Science & Engineering, Jiangxi Agricultural University, Nanchang 330045, China

**Keywords:** chicken meat, neomycin, chloramphenicol, surface-enhanced Raman spectroscopy, principal component analysis, linear discriminant analysis

## Abstract

In the process of chicken breeding, there has been a great deal of abuse of antibiotics. Antibiotics can enter the human body along with the chicken meat, comprising a possible risk to human health. In this paper, principal component analysis (PCA)–linear discriminant analysis (LDA) was chosen to classify neomycin (NEO) and chloramphenicol (CAP) residues in chicken meat. A total of 400 chicken meat samples were used for the classification, of which 268 samples and 132 samples were used as the training sets and the test sets, respectively. The experimental condition of SERS spectrum collection was optimized, including the use of a gold colloid and active agent, and an improvement in the adsorption time. The optimal measurement conditions for the SERS spectra were an adsorption time of 4 min and the use of a 14th-generation gold colloid as the enhanced substrate without a surfactant. For three groups of different spectral preprocessing methods, the classification accuracies of PCA-LDA models for test sets were 78.79% for baseline correction, 84.85% for the second derivative and 100% for the second derivative combined with baseline correction. LDA was used to establish a classification model to realize the quick determination of NEO and CAP residues in chicken meat by SERS. The results showed that the characteristic peaks at 546 and 666 cm^−1^ could be used to distinguish NEO and CAP residues in chicken meat. The classification model based on PCA-LDA had higher classification accuracy, sensitivity and specificity using a second derivative combined with baseline correction as the spectral preprocessing method, which shows that the SERS method based on PCA-LDA could be used to perform the classification of NEO and CAP residues in chicken meat quickly and effectively. It also verified the feasibility of PCA-LDA to effectively classify chicken meat samples into four types. This research method could provide a reference for the measurement of such antibiotic residues in chicken meat in the future.

## 1. Introduction

Antibiotics, also known as antimicrobials, are a kind of secondary metabolites produced by microorganisms (including bacteria, fungi, and actinomyces) or higher plants and animals in the course of life, with anti-pathogenic or other activities [1,2]. There has been a great deal of abuse of antibiotics in the process of chicken breeding [3]. Antibiotics can enter the human body via chicken meat and accumulate in the body, thus endangering human health, such as by causing damage to the human immune system, serious adverse drug reactions, and disease [4,5]. Bacterial resistance has gradually increased, with the time interval becoming shorter and shorter [6,7]. Although there have been clear regulations on the use of antibiotics, abuse of antibiotics still exists [8,9]. In order to prevent antibiotics from entering the human body with excessive chicken meat, it is particularly essential to find an efficient, quick and simple method for the determination of antibiotic residues in chicken meat [10,11]. Neomycin (NEO) and chloramphenicol (CAP) belong to the aminoglycoside and chloramphenicol group of antibiotics [12,13], respectively. They have inhibitory effects on many bacteria and play an important effect in the curing of bacterial infections caused by typhoid bacilli, escherichia coli and influenza bacilli. At present, common methods for the determination of antibiotic residues in chicken meat at home and abroad are high-performance liquid chromatography, thin-layer chromatography, microbial detection and immunoassay [14,15]. These methods have the peculiarities of precision and high sensitivity, but the pretreatment process involved in them is tedious and high-cost, and has a slow detection speed [16,17], so it is not fit for on-site real-time quick detection and screening.

SERS technology is a new technology developed on the basis of the original Raman spectrum, which solves issue of Raman scattering of the substance being weak and difficult to detect; the former technology, on the other hand, has high sensitivity, has a high resolution and experiences little interference from water compared to traditional methods such as LC-MS, HPLC, or GC [18,19]. SERS technology coupled with active agents has been used in bioengineering, medicine [20], food detection and other fields [21,22], as well as in antibiotic residues in poultry meat [23,24]. The active substrate must have the following advantages: high stability, an obvious enhancement effect and good reproducibility. A Ag-TiO_2_ nanoparticle SERS-active substrate was used for the detection of five widely used quinolone antibiotics in actual water samples. The LOD for this is below the maximum of residue limit set by the European Union [6]. Teng et. al present a Raman sensor for the detection of quinolone antibiotic residues in a water environment based on Ag SERS substrate. It can effectively detect the Raman signal of unlabeled trace quinolone antibiotic residues. The ciprofloxacin and norfloxacin detection limits are 10^−10^ M and 10^−11^ M, respectively [25]. A porous anodized aluminum oxide (AAO) coated with silver nanofilms was developed as a SERS-active substrate. It was used to detect trace levels of chloramphenicol, with an enhancement factor of approximately 2 × 10^4^, and the detection limit for chloramphenicol was 7.5 ppb [26]. The deep learning model for the detection and classification of antibiotics based on plasma-treated bimetallic nanofibers was used as a sensitive SERS platform. The very good applicability of the designed SERS substrate was realized for the detection of two antibiotics, fluconazole and lincomycin [27]. Penicillin G sodium, amoxicillin and chloramphenicol residues in poultry meat were detected using SERS detection technology. The method had a lower detection limit and a higher recovery rate [28]. Combined with the high sensitivity of SERS and the convenience of the gold-standard strip, a new SERS method was established to detect residual neomycin, lincomycin and quinolone antibiotics in milk, achieving the ultra-sensitive detection of a single antibiotic or multiple antibiotics [29]. A rapid quantitative analysis model was established for spiramycin, tilmicosin and kitasamycin aqueous solution using the SERS detection technique. The characteristic peaks of the three antibiotics in dairy products were determined [30]. Many researchers used have different methods to study antibiotic residues in agricultural products [31,32]. At present, there is no study on the identification of NEO and CAP residues in chicken meat by SERS

Based on this, SERS technology was employed to expedite the detection of NEO and CAP residues in chicken meat in this study. The SERS detection parameters for the detection of NEO and CAP residues in chicken meat were optimized by using single-factor experiments with a gold colloid and an active agent, and considering the adsorption time. The original spectral data were preprocessed using a second derivative combined with baseline correction, and the feature vectors were extracted by PCA. LDA was used to establish a classification model to realize the quick determination of NEO and CAP residues in chicken meat. This research method could act a reference for the determination of similar antibiotic residues in chicken meat in the future.

## 2. Materials and Methods

### 2.1. Reagents and Chemicals

The instruments used were as follows: Micro Raman Spectrometer (DXR^TM^, Saimer Feshir Technology Co., Ltd., Fuzhou, China); Ultrasonic Cleaner (JK-50B, Hefei Jinnik Machinery Manufacturing Co., Ltd., Hefei, China); Electronic Balance (FA1004B, Beijing Kewei Yongxing Instrument Co., Ltd., Beijing, China); Intelligent Constant Temperature Magnetic Mixer (ZNCL-T, Zhengzhou Yarong Instrument Co., Ltd., Zhengzhou, China); Vacuum Freeze Dryer (FD-1A-50, Beijing Boyikang Test Instrument Co., Ltd., Beijing, China).

The materials and substances used were as follows: tetrachloroauric acid trihydrate (≥49.0%, Sigma-Aldrich Trading Co., Ltd., Shanghai, China); NEO standard substance (purity 99.4%, Shanghai Aladdin Biochemical Technology Co., Ltd., Shanghai, China); CAP standard substance (purity 98%, Shanghai Aladdin Biochemical Technology Co., Ltd., Shanghai, China); sodium citrate, sodium chloride, magnesium sulfate, and β-cyclodextrin (analytically pure, Xilong Chemical Co., Ltd., Shantou, China); chicken meat (purchased from experimental stations in Jiangxi Agricultural University).

### 2.2. Preparation of Samples

For the preparation of NEO and CAP standard solution, 25 mg NEO or CAP standard substance was dissolved in 500 mL ultra-pure water to obtain NEO or CAP standard solution in an amount of 50 mg/L, respectively. The standard solution was used to prepare other different concentrations of NEO solution, CAP solution and a solution of NEO coupled with CAP several times.

For the chicken meat pretreatment, firstly, the chicken meat samples without any antibiotics stored in the refrigerator at −20 °C for 5 h were sliced with a slicer, and dried in a vacuum freeze dryer for 48 h; then, they were taken out and set aside. Secondly, the dried chicken meat was soaked in different concentrations of NEO solution, CAP solution and the solution NEO coupled with CAP, and dried at room temperature. Lastly, four groups of chicken meat samples, including the chicken meat samples containing NEO, CAP and NEO coupled with CAP residues and blank chicken samples without NEO and CAP residues, were prepared respectively.

For the preparation of the gold colloid, the enhancement of the SERS signal was closely related to the substrate, and the activity of the substrate was the key factor affecting the enhancement effect of SERS [33]. The active substrate must have the advantages of high stability, an obvious enhancement effect and good reproducibility. In this study, two methods were used to prepare five gold colloids with different particle sizes: (1) when the HAuCl_4_ solution was boiling, 3.7 mL of 1% sodium citrate solution was quickly added to obtain gold colloids. Four kinds of gold colloids with different particle sizes were prepared by adding different amounts of sodium citrate solution [34]. (2) A 14th-generation gold colloid was prepared using the Neus G. Bastús preparation method. The gold colloid with a gradually increasing particle size was obtained by constantly adding chloroauric acid solution and sodium citrate solution. A round-bottom flask containing 150 mL of 2.2 mM sodium citrate solution was heated in an intelligent constant-temperature magnetic stirrer and stirred vigorously for 15 min. When the solution was boiled, 1 mL of 25 mM HAuCl_4_ solution was added. After the HAuCl_4_ solution was added, the color of the mixed solution changed from yellow to blue-gray. After 10 min, it turned pale pink, and the resulting gold particles were coated with charged citric acid ions and suspended in water. The resulting gold particles were immediately cooled in the same container until the solution temperature reached 90 °C. The cooled gold particles were added to 1 mL sodium citrate solution at a concentration of 60 mM, and then added to 1 mL of HAuCl_4_ at a concentration of 25 mM after 2 min. The resulting gold colloid was characterized after 30 min. It was possible to prepare the gold colloid with an increasing size by repeating this process13 times [35]. The prepared gold nanoparticles were then stored at 4 °C away from light.

### 2.3. Collection of Raman Spectra

The parameters of the micro-Raman spectrometer were as follows: a laser energy of 10 mW, an acquisition exposure time of 5 s, a preview acquisition time of 10 s, a sample exposure resolution of 10 times, and a background exposure resolution of 16 times. An appropriate amount of gold colloid was added on the pretreated chicken meat sample that had been put on the slide, and then the slide was put on top of a glass table. After adsorption for a certain period of time, the Raman spectrum was collected by a DXR^TM^ micro-Raman spectrometer with a 785 nm laser wavelength. SERS spectra for each sample were collected 5 times, and the SERS spectrum of the sample as the average spectrum.

### 2.4. Optimization of Test Conditions

The optimization of test conditions included the selection of the gold colloid type, the use of an active agent and an improvement in the adsorption time. SERS spectra were collected by the DXR^TM^ micro-Raman spectrometer. SERS spectra for each sample were collected 5 times, and the SERS spectrum of the sample was obtained as the average spectrum. The SERS signal characteristic peak intensities were compared at 546 and 666 cm^−1^.

For the optimization of the gold colloids, a gold colloid was prepared by adding 3.7 mL sodium citrate and a 14th-generation gold colloid was also used; these were added to the chicken meat containing NEO and CAP on a glass slide to adsorb for 4 min. Then, the glass slide was placed on the glass platform to measure the SERS spectra.

For the optimization of active agents, we added salts like sodium chloride or potassium chloride, and the salt ions were able to shield the charges on the surface of the nanoparticles, reducing electrostatic repulsion between particles and promoting aggregation to form ‘hot spots’. The 14th-generation gold colloids mixed with different active agents (NaCl solution, magnesium sulfate solution and cyclodextrin solution) and without an active agent were dripped onto the chicken meat containing NEO and CAP on the glass slide to adsorb for 4 min Then, the glass slides were placed on the glass platform to measure the SERS spectra.

For the optimization of adsorption time, the 14th-generation gold colloid was added to the chicken meat containing NEO and CAP on the glass slides, and then the glass slide was placed on the glass platform. Their Raman spectra were collected when the adsorption times were 1, 2, 4, 6, 8 and 10 min respectively.

### 2.5. Data Analysis

The Raman spectrum was affected by many factors in the process of collection, such as light, temperature, and sound, which affected the spectral quality [36]. In this study, different spectral preprocessing methods including baseline correction, a second derivative and baseline correction combined with a second derivative were used to preprocess the original data in order to eliminate noise and interference while retaining the main information. PCA is a multivariate statistical method that converts multiple indicators into several comprehensive indicators [37]. The feature vector was extracted by PCA for reducing the deviation as much as possible. LDA is an effective qualitative analysis method which achieves the accuracy of discriminant analysis by reducing the proportion of intra-class variance. LDA was used to establish a classification model for the rapid identification of NEO and CAP residues in the chicken meat. All data analysis was based on the MATLABR2010a and SPSS21.0 platform.

## 3. Results and Discussion

### 3.1. Analysis of SERS Spectral Characteristics of NEO and CAP Residues in Chicken

**Figure 1:**
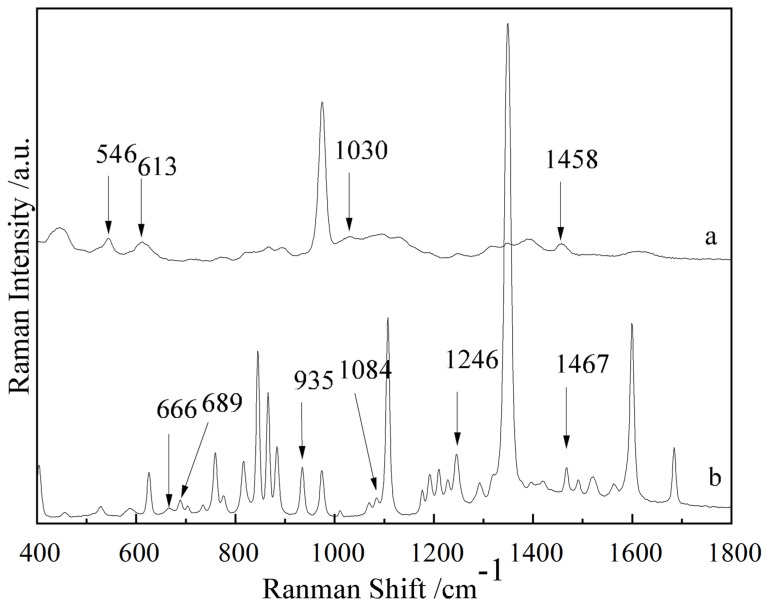
The Raman spectra of the NEO substance (a) and CAP substance (b).

**Figure 2:**
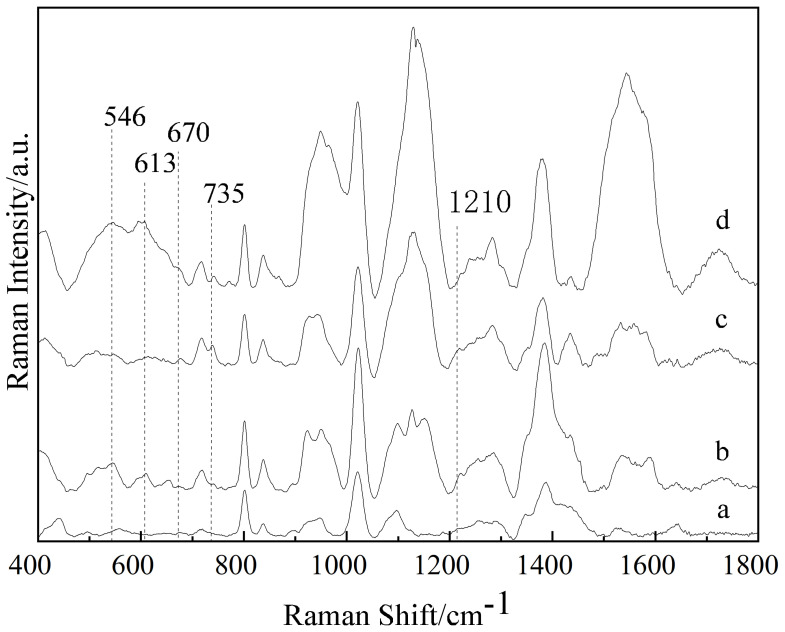
The SERS spectra of the gold colloid (a), NEO aqueous solution with 10 mg/L (b), CAP aqueous with 10 mg/L (c) and aqueous solution of NEO coupled with CAP (d).

The Raman spectra of the NEO and CAP standard substances were collected, and the results are shown in Figure 1. The SERS spectra of the gold colloids, NEO aqueous solution with 10 mg/L, CAP aqueous solution with 10 mg/L and aqueous solution of NEO coupled with CAP were collected, and their SERS spectra are shown in Figure 2. From curve (a) in Figure 1, it can be seen that the NEO standard substance had obvious characteristic peaks at 546, 613, 1030 and 1458 cm^−1^. Based on curves (b) and (d) of Figure 2, the SERS spectra of NEO aqueous solutions had distinct characteristic peaks at 546 and 613 cm^−1^. Therefore, the characteristic peaks at 546 and 613 cm^−1^ could be used to determine whether NEO residues were present in the solution.

From curve (b) in Figure 1, it could be seen that the CAP standard substance had distinct characteristic peaks at 666, 689, 935, 1084, 1246 and 1467 cm^−1^. From curve (c) and (d) in Figure 2, it could be observed that there were obvious characteristic peaks at 670 and 735 cm^−1^ in the CAP solution, while there were no obvious characteristic peaks at 670 and 735 cm^−1^ on curve (a) and curve (b) in Figure 2. The CAP standard substance had an obvious characteristic peak at 666 cm^−1^, while the CAP aqueous solution had an obvious characteristic peak at 670 cm^−1^. This phenomenon may be due to the plasma resonance between the CAP solution and gold colloid, and the peak had a red shift from 666 cm^−1^ to 670 cm^−1^. Therefore, the characteristic peaks at 670 and 735 cm^−1^ could be used to determine whether CAP residues were present in the solution.

**Figure 3:**
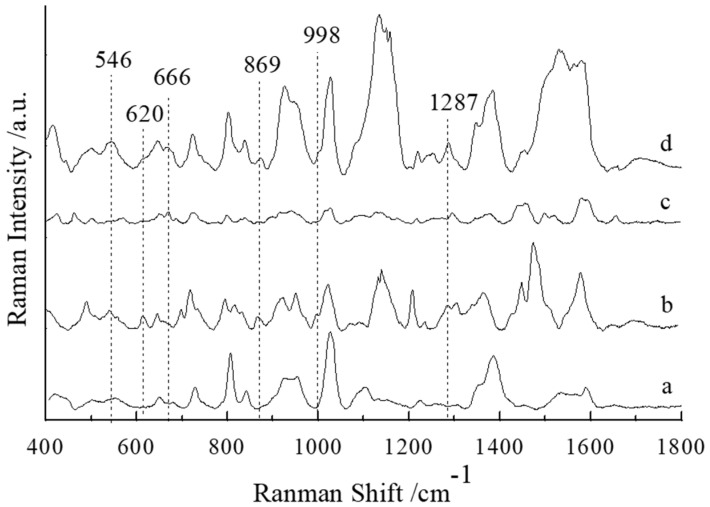
The SERS spectra of the blank chicken meat (a), chicken meat containing 10 mg/kg NEO (b), chicken meat containing 10 mg/kg CAP (c) and chicken meat containing NEO coupled with CAP (d).

The SERS spectra of blank chicken meat, chicken meat containing NEO, chicken meat containing CAP and chicken meat containing NEO coupled with CAP residues were collected and are shown in curve (a)~(d) in Figure 3. Comparing curve (a), (b), (c) and (d), the SERS spectra of chicken meat containing NEO coupled with CAP had an obvious characteristic peak at 546, 620, 666, 869, 998 and 1287 cm^−1^, while the SERS spectrum of blank chicken meat had no characteristic peaks at these positions, so these peaks may be characteristic peaks of NEO and CAP. The SERS spectrum of chicken meat containing NEO showed that there were obvious characteristic peaks at 546, 620, 869, 998 and 1287 cm^−1^, but the SERS spectrum of blank chicken meat had no obvious characteristic peaks at these positions, so according to the SERS spectra of chicken meat containing NEO and chicken meat containing NEO coupled with CAP, the characteristic peaks at 546, 620, 869, 998 and 1287 cm^−1^ may be the characteristic peaks of NEO in chicken meat. However, it can be seen from Figure 1 and Figure 2 that the characteristic peaks of NEO standard solution were 546, 613, 1030 and 1458 cm^−1^, and those of NEO aqueous solution were 546 and 613 cm^−1^, so it could be determined that the peak at 546 cm^−1^ was the characteristic peak of NEO in chicken meat. The SERS spectra of chicken meat containing CAP and chicken meat containing NEO coupled with CAP show that there were obvious characteristic peaks at 666 cm^−1^, while the SERS spectrum of blank chicken meat had no characteristic peaks at these positions. At the same time, compared to Figure 1 and Figure 2, the peak at 666 cm^−1^ was the characteristic peak of CAP in chicken meat. Therefore, the characteristic peaks at 666 cm^−1^ and 546 cm^−1^ could indicate the presence of CAP and NEO residues in chicken meat, respectively.

### 3.2. Optimization of SERS Detection Conditions

**Figure 4:**
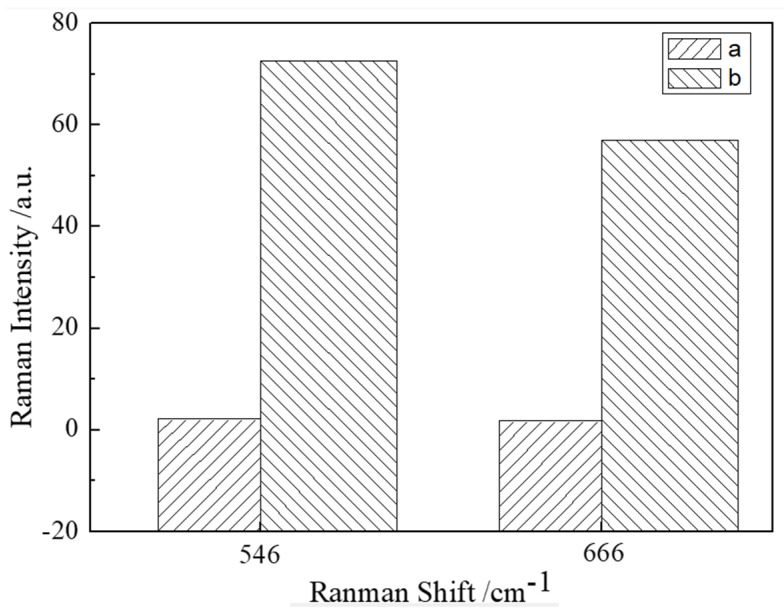
Effect of gold colloid type on SERS spectra: (a) the gold colloid prepared with 3.7 mL sodium citrate and (b) the gold colloid of the 14th generation.

Different enhanced substrates had different enhancement effects on the same substance. In this study, the Raman spectra of chicken meat containing NEO coupled with CAP residues were collected using a gold colloid prepared with 3.7 mL sodium citrate and a 14th-generation gold colloid as enhanced substrates. Figure 4 shows the SERS spectral intensities at 546 and 666 cm^−1^ of NEO coupled with CAP residues in chicken meat when two different kinds of gold colloid were added. It can be seen that when the 14th-generation gold colloid was used as the enhanced substrate, the intensities of the characteristic peaks at 546 and 666 cm^−1^ were significantly higher than the SERS spectral intensities of the gold colloid prepared by adding 3.7 mL sodium citrate. Therefore, the 14th-generation gold colloid was used as the SERS enhancement substrate in this study.

**Figure 5:**
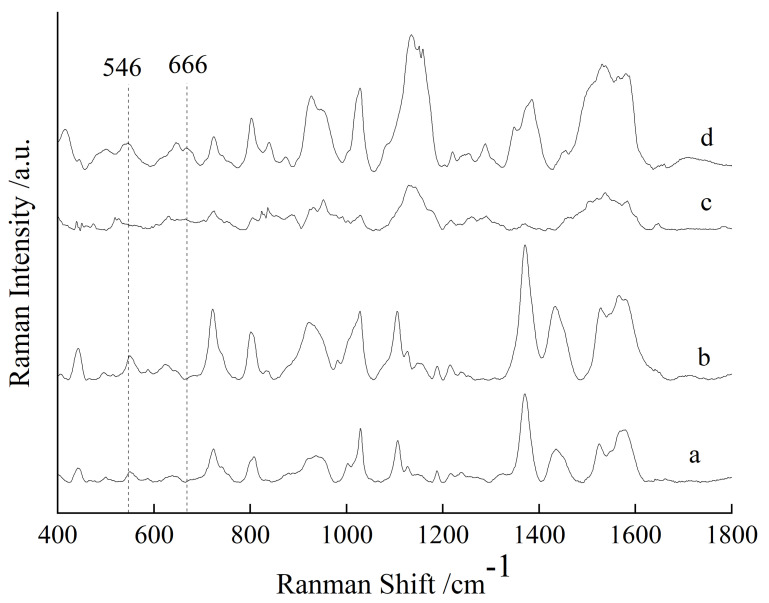
Effect of different activators on SERS spectra: (a) NaCl solution, (b) magnesium sulfate solution, (c) cyclodextrin solution, and (d) no active agent.

In addition to the enhanced substrates, active agents can also influence the signal intensity of SERS. Upon adding salts like sodium chloride or potassium chloride, the salt ions can shield the charges on the surface of the nanoparticles, reducing electrostatic repulsion between the particles and promoting aggregation to form ‘hot spots’. The SERS spectra of chicken meat containing NEO and CAP with NaCl solution, magnesium sulfate solution and cyclodextrin solution as active agents are shown in Figure 5. Compared to the four curves in Figure 5, the Raman characteristic peak at 546 cm^−1^ of NEO is clearly displayed on curves (a), (b) and (d) in Figure 5, while it does not appear on curve (c) in Figure 5. The Raman characteristic peak at 666 cm^−1^ of CAP only appeared on the curve (d) in Figure 5, but not in the other three curves. The Raman characteristic peaks of CAP in chicken meat could not be collected when NaCl solution, magnesium sulfate solution and cyclodextrin solution were used as the active agents, but they could be collected when the active agents were not added. This phenomenon may be due to the combination of active agent molecules, i.e., gold colloid and NEO or CAP molecules, affecting the enhancement properties of the gold colloid. Because the intensity of the SERS signal under the condition in which there was no active agent was stronger than that under the condition in which there was an active agent, we did not choose to an the active agent to the enhanced substrates in this study.

**Figure 6:**
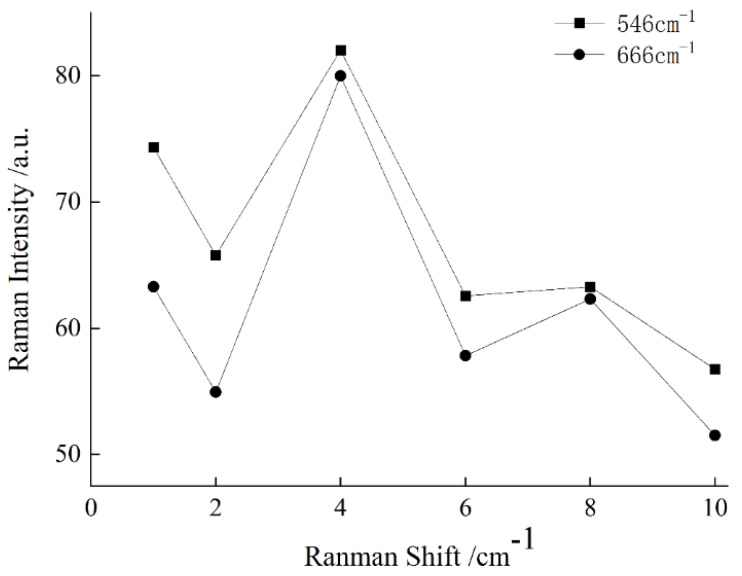
Effect of adsorption time on SERS signal.

A change in the adsorption time will affect the aggregation state of gold colloids and NEO or CAP molecules, and the intensity of the SERS signal will change with the change in the aggregation state. Therefore, the intensity of the SERS spectra can be affected by the adsorption time. The SERS spectral intensities of chicken meat containing NEO coupled with CAP residues at 546 and 666 cm^−1^ are shown in Figure 6 at different adsorption times (1, 2, 4, 6, 8 and 10 min). It could be seen that the SERS signal intensities at 546 and 666 cm^−1^ were the strongest when the adsorption time was 4 min. Therefore, the optimal adsorption time selected in this study was 4 min.

### 3.3. Selection of Spectral Preprocessing Methods

Because the Raman spectrum could be interfered with by external factors, there was a lot of interference information in the Raman spectrum. In order to eliminate the effect of these interference factors, three spectral preprocessing methods were compared: baseline correction, a second derivative and baseline correction combined with a second derivative. Chicken meat samples containing NEO, CAP, and NEO coupled with CAP and blank chicken meat samples were treated as groups I, II, III, and IV, respectively. Table 1 lists the classification accuracy of PCA-LDA models for test sets using the three spectral preprocessing methods.

As seen from Table 1, for the three different kinds of spectral preprocessing methods, the classification accuracies of PCA-LDA model for the test sets were 78.79%, 84.85% and 100%, respectively. Among them, baseline correction combined with a second derivative as the spectral preprocessing method had the highest classification accuracy, which was 100%. The results showed that the four kinds of chicken meat samples could be classified and identified completely accurately using baseline correction coupled with second derivative as the spectrum preprocessing method. Therefore, baseline correction coupled with a second derivative was selected as the spectral preprocessing method in this study.

### 3.4. Feature Parameter Extraction Based on PCA

**Figure 7:**
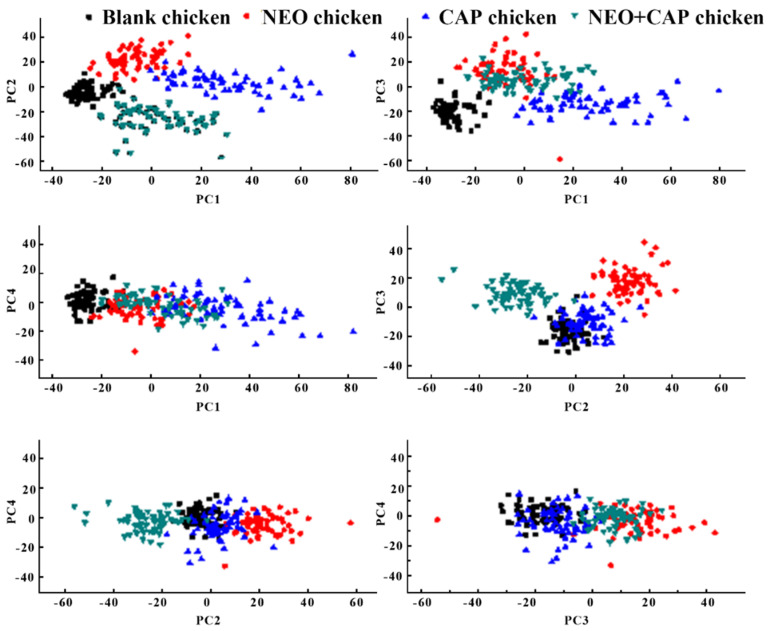
PCA score plots of first four principal components of training set.

The SERS spectra after spectral preprocessing were analyzed by PCA, and the PCA score diagrams of the first four principal components were drawn, as shown in Figure 7. The PCA score maps of PC1 and PC3 could be used to distinguish blank chicken meat samples from the other three types of samples. The PCA score maps of PC2 and PC3 could also be used to distinguish chicken meat samples containing NEO coupled with CAP from the other three types of samples. However, chicken meat samples containing NEO or CAP were difficult to distinguish from other samples based on the PCA score chart. According to the PCA score map of the first four principal components of the training set, these four types of samples could not be completely distinguished. Therefore, the linear discriminant analysis method was applied to expand the differences between these four types of sample data in order to better distinguish and effectively separate them in this study.

### 3.5. Classification Results of NEO and CAP Residues in Chicken Meat Based on LDA

**Figure 8:**
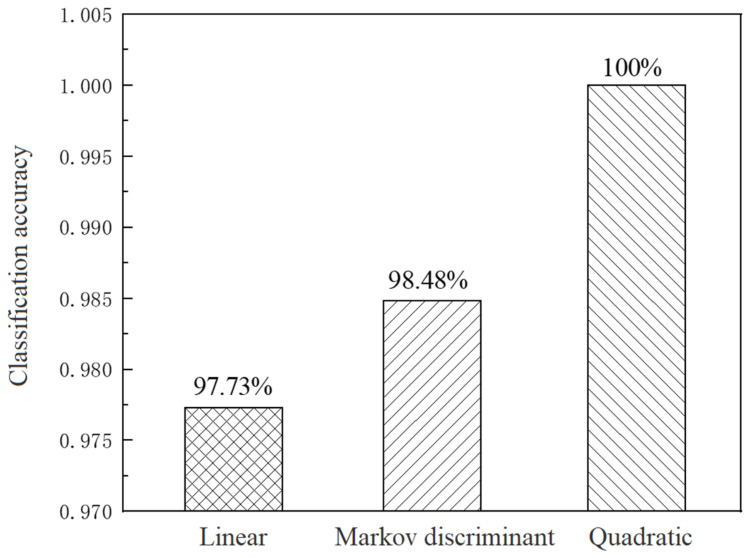
Classification accuracy of NEO coupled with CAP residues in chicken meat for test set using three option methods based on PCA-LDA.

In this study, LDA was used to classify four types of chicken meat samples. In the process of establishing the PCA-LDA classification model, the different discriminant functions would obtain the classification accuracies. Figure 8 shows the classification accuracies of the test set achieved by LDA combining three different discriminant functions. Among them, the classification accuracy obtained by using the quadratic function as the discriminant function was higher, up to 100%, compared to that in previous research, so there has been an improvement in accuracy. Therefore, the quadratic function was chosen as the discriminant function of LDA in this study.

The PCA-LDA classification models coupled with three spectral preprocessing methods were established. The sensitivities and specificities of the four types of chicken meat for the test set are displayed in Table 2. Using baseline correction as a spectrum pretreatment method, the sensitivity of blank chicken meat samples was highest (96.97%), and the specificity of chicken meat samples containing NEO was highest (97.98%). Using the second derivative as a spectrum pretreatment method, the sensitivity of the chicken meat samples containing CAP was 98.99%, and those of the other three types were 100%. In addition, the specificity of the chicken meat samples containing CAP was the highest (99.02%). Using baseline correction combined with a second derivative as spectral preprocessing method, the sensitivities and specificities of the four samples for the test set were all 100%. Compared with the first two spectral preprocessing methods, this spectral preprocessing method could make the differences between the four types of chicken meat samples more obvious, and therefore the sensitivity and specificity of the samples has been improved.

After the SERS spectra of the chicken meat samples were preprocessed by using baseline correction coupled with a second derivative, the classification model established by PCA combined with LDA had higher classification accuracy, sensitivity and specificity. This shows that spectral preprocessing method based on baseline correction coupled with a second derivative could more quickly and effectively achieve the classification of NEO and CAP residues in chicken, and it also verifies the feasibility of using PCA-LDA to quickly and effectively identify samples, such as the four types of chicken meat samples used in this study.

## 4. Conclusions

The SERS method has high sensitivity, the ability for rapid detection, and the potential for multi-component analysis in food antibiotic testing. The current SERS system is less effective than established techniques such as LC-MS, HPLC, or the GC detection level, though it is more portable and has a shorter detection time. In this paper, using a gold colloid as a reinforced substrate, SERS technology and PCA-LDA were used to classify NEO and CAP residues in chicken meat. The Raman characteristic peaks of a standard antibiotic substance, an aqueous solution and chicken meat were analyzed; the peak at 546 cm^−1^ was used to confirm whether there were NEO residues in chicken meat, and the peak at 666 cm^−1^ was used to confirm whether there were CAP residues in chicken meat. In the process of identifying NEO and CAP residues in chicken meat, baseline correction coupled with a second derivative was used as the spectral preprocessing method, and the classification accuracy reached 100%. A classification model was established based on PCA-LDA, and the first four scores of PCA were used as the input values of the LDA classification model to classify four types of chicken meat samples. The sensitivity and specificity of the classification model was able to reach 100%. The classification model established via PCA combined with LDA had higher classification accuracy, sensitivity and specificity, which shows that using baseline correction combined with a second derivative as a spectral preprocessing method could more effectively realize the classification of NEO and CAP residues in chicken meat. It also verifies the feasibility of using PCA-LDA to quickly and effectively identify samples such as the four types of chicken meat samples used in this study.

## Figures and Tables

**Table 1:** Classification results of NEO and CAP residues in chicken meat for test sets by different spectral pretreatment methods based on PCA-LDA.

Pretreatment Method	Group	Classified As				Accuracy
		I	II	III	IV	
Baseline correction	I	26	0	3	4	78.79%
	II	0	25	5	3	
	III	2	3	21	7	
	IV	0	0	1	32	
Second derivative	I	33	0	0	0	84.85%
	II	3	30	0	0	
	III	0	1	31	1	
	IV	0	0	0	33	
Baseline correction combined with second derivative	I	33	0	0	0	100%
	II	0	33	0	0	
	III	0	0	33	0	
	IV	0	0	0	33	

**Table 2:** Sensitivity and specificity of PCA-LDA models established by different spectral pretreatment methods for test set.

Sample	Baseline Correction		Second Derivative		Baseline Correction Combined with Second Derivative	
	Sensitivity	Specificity	Sensitivity	Specificity	Sensitivity	Specificity
I	78.79%	97.98%	100%	96.97%	100%	100%
II	75.76%	90.91%	98.99%	99.02%	100%	100%
III	63.63%	93.94%	100%	86.73%	100%	100%
IV	96.97%	85.86%	100%	98.99%	100%	100%

## Data Availability

The data collected in this research is available upon request.
